# Leading edge competition promotes context-dependent responses to receptor inputs to resolve directional dilemmas in neutrophil migration

**DOI:** 10.1016/j.cels.2023.02.001

**Published:** 2023-02-23

**Authors:** Amalia Hadjitheodorou, George R. R. Bell, Felix Ellett, Daniel Irimia, Robert Tibshirani, Sean R. Collins, Julie A. Theriot

**Affiliations:** 1 Department of Bioengineering, Stanford University, Stanford, CA, USA; 2 Department of Biology and Howard Hughes Medical Institute, University of Washington, Seattle, WA, USA; 3 Department of Microbiology and Molecular Genetics, University of California, Davis, Davis, CA, USA; 4 Department of Surgery, BioMEMS Resource Center, Massachusetts General Hospital, Harvard Medical School, Boston, MA, USA; 5 Department of Statistics and Biomedical Data Science, Stanford University, Stanford, CA, USA; 6 Present address: Chan Zuckerberg Biohub, San Francisco, CA, USA; 7 Lead contact

**Keywords:** neutrophils, motility, polarity, cytoskeleton, Cdc42, microfluidics, optogenetics, statistical learning

## Abstract

Maintaining persistent migration in complex environments is critical for neutrophils to reach infection sites. Neutrophils avoid getting trapped, even when obstacles split their front into multiple leading edges. How they re-establish polarity to move productively while incorporating receptor inputs under such conditions remains unclear. Here, we challenge chemotaxing HL60 neutrophil-like cells with symmetric bifurcating microfluidic channels to probe cell-intrinsic processes during resolution of competing fronts. Using supervised statistical learning, we demonstrate that cells commit to one leading edge late in the process, rather than amplifying structural asymmetries or early fluctuations. Using optogenetic tools, we show that receptor inputs only bias the decision similarly late, once mechanical stretching begins to weaken each front. Finally, a retracting edge commits to retraction, with ROCK limiting sensitivity to receptor inputs until the retraction completes. Collectively, our results suggest that cell edges locally adopt highly stable protrusion/retraction programs which are modulated by mechanical feedback.

## eTOC Blurb

Neutrophils navigating in complex tissue environments frequently split their leading edges into competing fronts that may try to pull the cell in different directions. We combined bifurcating microfluidic channels with optogenetic control of receptors to probe the mechanism of communication between the competing fronts. We found that the cells resolve their dilemma by coordinating activity of the edges, mediated via mechanical stretching.

## Graphical Abstract

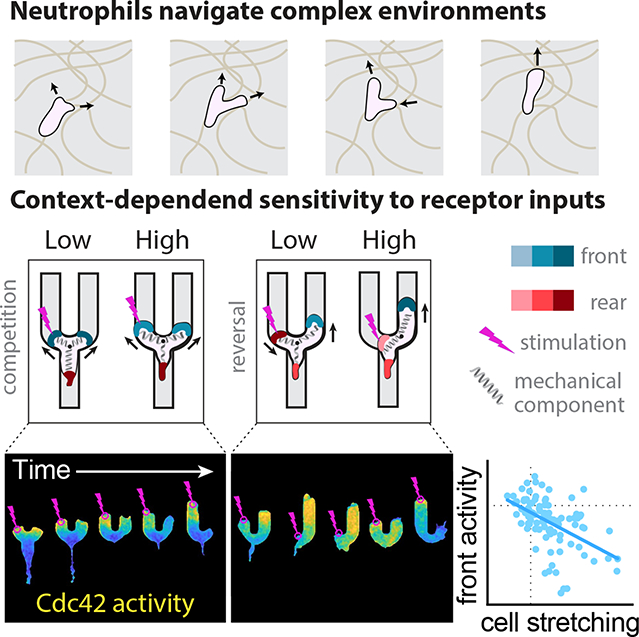

## INTRODUCTION

During immune surveillance, neutrophils use chemotaxis to navigate through complex tissue environments to reach sites of infection and inflammation. In addition to chemical cues, the migration paths of neutrophils are strongly influenced by physical constraints of the environment. *In vivo* imaging in zebrafish^1^ and mice^2,3^ has revealed that neutrophils chemotaxing in dense tissues often split their leading edges into multiple competing fronts, as they encounter physical barriers in their extracellular matrix. This splitting may create conflicts in which chemical gradients favor fronts leading towards dead-end paths. Nevertheless, neutrophils preserve their physical integrity and continue navigation by sustaining one front and abandoning the rest, without tearing themselves apart. How exactly this happens is not fully understood.

The cellular polarity circuit could play a central role in this process, as it coordinates cell motility by defining separate cell front and rear domains^4–8^. G-protein coupled receptors (GPCRs) that detect chemoattractants connect to the polarity circuit by signaling through G_i_α G-proteins to activate cell front signaling cascades^9^. Major targets include the small GTPases Cdc42 and Rac that act as master regulators at the cell front to organize polarity and promote protrusive behavior by mediating branched actin assembly^10^. At the cell rear, the small GTPase RhoA acts through the kinase ROCK and phosphorylation of myosin regulatory light chain to facilitate assembly of myosin II filaments and actomyosin contraction, which is responsible for the retraction of the cell’s trailing edge^11^. Locally, the cell front (protruding) and rear (retracting) domains are self-reinforcing via positive feedback and are mutually antagonistic^6,11–13^. Indeed, modeling has demonstrated that this signaling architecture is sufficient to generate stable cell polarization in response to external cues or even spontaneously by amplifying molecular noise^14–17^.

Neutrophils are exquisitely sensitive to external cues from their environment, which is important for their *in vivo* migration. Previous work has explored how this sensitivity can bias choices between competing fronts. Specifically, experiments using microfluidic channels have shown that neutrophils can make decisions to favor the competing front oriented toward the stronger chemical signal^18,19^. Neutrophils are also able to distinguish between alternative paths with different physical parameters, including favoring the path with lowest hydraulic resistance^20^ or largest diameter^21^. However, in these studies, external cues could not be changed dynamically while concurrently monitoring internal cell processes, and so it remains unclear whether or how these external cues interact with the cell’s intrinsic polarity circuit to resolve competing fronts.

To understand how external inputs and the polarity circuit contribute to resolution of competing cell fronts, it is first critical to determine whether early or pre-existing cellular asymmetry could act as a hidden variable that influences the cell’s directional decision. Supporting this hypothesis, in the studies described above, directional preferences were never totally deterministic, but instead emerged as a quantitative bias toward the preferred path^18–21^. The source of pre-existing asymmetry could be small fluctuations at the level of molecular noise or structural inequalities between the two competing leading edges. For example, one leading edge might have a higher density of actin filaments or higher Cdc42 activity. The relevant asymmetry might not be the motility machinery itself; for example, the positioning of the nucleus or the microtubule organizing center have been shown to act as determinants of directional decision-making in dendritic cells^21^. In either of these cases, pre-existing structural, cytoskeletal or signaling asymmetries between the competing fronts would be predictive of the eventual directional outcome, even if the possible paths are otherwise equivalent.

Cell-intrinsic processes could also play a critical role by coordinating the behavior of competing fronts. One hypothesis is that biochemical feedback loops of the cellular polarity circuit could promote resolution by amplifying asymmetry, regardless of whether the asymmetry is pre-existing or spontaneously generated^13,15,22^. In this model, signaling and cytoskeletal activity would be expected to diverge at the competing fronts, with activity increasing or persisting at the eventual winning front and decreasing in the other(s). Another hypothesis involves the long-standing “local excitation / global inhibition” or “LEGI” model, which proposes that a rapidly diffusing negative signal downstream of receptors allows comparison of GPCR inputs in different parts of the cell^23,24^. The LEGI model would predict that an increase in receptor activity at one of the competing fronts would trigger a local increase in protrusion-promoting signaling and a simultaneous decrease in protrusive signaling at the other competing front(s). A final hypothesis involves mechanical coupling, which, through membrane or cytoskeletal tension, could inhibit protrusion at a distance to promote the formation of only a single cell front^25,26^. Mechanical coupling would be expected to weaken each front in a manner dependent on cell stretching, which could mediate competition until one front begins to retract. It is not yet known which (if any) of these mechanisms might be primarily responsible for the resolution of competing fronts formed by splitting when neutrophils encounter obstacles.

**Figure 1:**
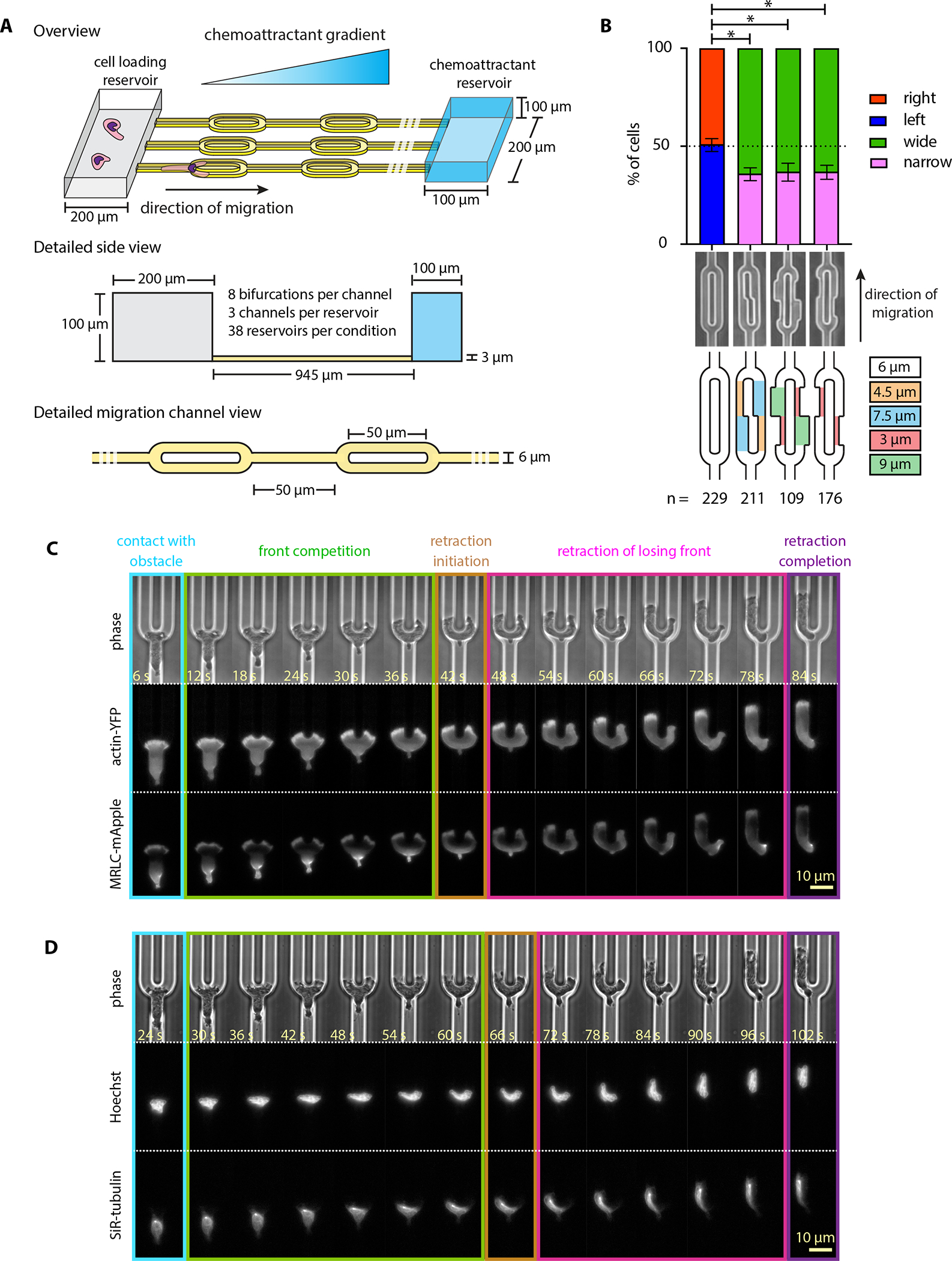
Cells choose directions randomly in an engineered symmetric environment **(A)** Schematic illustration of the microfluidic device engineered to probe symmetric decision-making of HL60 cells. **(B)** Stacked bar plots of the percentage of cells that turned left vs. right (symmetric design) or wide vs. narrow (for all other channel designs). Error bars indicate standard deviations assuming a binomial distribution around the cumulative mean of each design. Two-sided Fisher exact tests revealed significant differences between the symmetric design and the asymmetric designs (* p < 0.05). **(C-D)** Live-cell imaging snapshots of single HL60 cells expressing actin-YFP and MRLC-mApple **(C)** and of the cell nucleus (stained with Hoechst) and microtubules (stained with SiR-tubulin), **(D)** during symmetric decision-making, representatives of 164 and 169 individual cells respectively (Supplemental Movies 1–2). Images were captured every 3 s and subsampled for illustration purposes. Scale bar: 10 μm.

In this work, we developed an experimental system to dissect the cell-intrinsic processes leading to resolution of competing leading edges. A major difficulty for studying this process and testing the hypotheses described above is that the process is intrinsically noisy, with considerable cell-to-cell variability. We addressed this challenge by standardizing our experiments and automating protocols to obtain large datasets with highly reproducible conditions. To eliminate confounding extrinsic biases and cleanly resolve cell-intrinsic processes, we challenged HL60 neutrophil-like cells with symmetrically bifurcating microfluidic channels containing a simplified oval-shaped obstacle (Fig. 1A).

We first tested whether pre-existing asymmetries in cellular structure determine the directional outcomes. We used fluorescent reporters for primary cytoskeletal and structural features and designed an agnostic statistical learning approach to determine whether and at what stage in the process asymmetry in these structures is predictive of the turning direction.

We then addressed the question of coordination between the leading edges by introducing optogenetic and imaging tools that allowed us to activate receptor inputs with exquisite spatial and temporal control while monitoring polarity signaling directly. By monitoring polarity signaling changes over time in competing cell fronts, with and without receptor inputs, we tested predictions of the biochemical feedback, LEGI, and mechanical models. Furthermore, we modulated the timing of receptor activation to determine whether early asymmetries could be maintained or amplified to affect the ultimate outcome, or whether early inputs might have little effect because each cell front is strongly committed to its protrusive state until late in the decision-making process. To further probe the strength of local commitment to protruding versus retracting behaviors, we also used our optogenetic tools to ask whether local receptor activation could rescue a losing front and reprogram it to start protruding again. Taken together, our data suggest a context-dependent response to receptor inputs, whereby both active cell fronts and cell rears have strong commitment to their current fate, and coordination between competing fronts is largely mediated through long-distance mechanical interactions.

## RESULTS

### Cells choose directions randomly in an engineered symmetric environment

First, we sought to engineer a symmetric, tightly controlled and highly reproducible assay to analyze the resolution of two competing protrusive fronts of HL60 cells. Our microfluidic device consists of an array of migration channels that connect a shared loading reservoir and chemoattractant chambers (Fig. 1A). The chemoattractant gradient is established along the migration channels via diffusion (see Methods). Cells enter the migration channels following a 100 nM fMLF gradient. The migration channels are 6 μm in width and 3 μm in height, forcing HL60 cells to occupy the entire cross-section, so that cells are mechanically confined and able to move only along the axis of the channels. Symmetric oval-shaped bifurcations are positioned every 50 μm (Fig. 1A). We found that cells presented with this symmetric bifurcation turned with equal probability to the left and to the right (Fig. 1B).

Cells responded to contact with the obstacle by splitting their front into two, with one active protrusion extending into each branch of the bifurcation. These two fronts competed, and the competition phase was resolved when one front started retracting, allowing the other side to “win” and guide the cell around the obstacle.

To gain better insight into how cell behavior depends on the mechanical constraints, we sought to quantify how directional bias changes as a function of channel geometry. To that end, we designed channels that have one wider and one narrower branch either immediately at the obstacle apex (2nd design from the left Fig. 1B) or at a 4 μm distance past the apex (3rd and 4rth designs from the left Fig. 1B). In designing channel geometries (Fig. 1B), we matched the hydraulic resistance between the two bifurcating branches, and only considered events where migrating cells did not have any other neighboring cells (preceding or following them) in the same channel. Cells showed a modest (~ 63%) but consistent preference for the initially wider branch, regardless of whether the asymmetry was introduced in the immediate vicinity of the obstacle apex or further away, suggesting that decisions are not finalized until cells have extended several micrometers into each channel. The preference of HL60 cells for wider channel branches is reminiscent of that of dendritic cells for larger sized channels^21^.

We then returned to the symmetric case to analyze which cellular asymmetries contribute to the resolution of competing fronts and when they arise. For many kinds of symmetry-breaking, stochastic fluctuations in the system create small asymmetries that may be amplified to determine the outcome. However, in the case of a migrating cell, fluctuations will occur for multiple different components, and the polarity circuit could buffer some and amplify others. We thus used fluorescent reporters for primary cytoskeletal and structural features to determine whether fluctuations in the asymmetry of any of these components might determine directional outcomes. Specifically, we used a neutrophil-like HL60 cell line stably expressing both actin fused to YFP and myosin regulatory light chain (abbreviated as MRLC) fused to mApple, and observed actin and myosin localization dynamics during decision-making (Supplemental Movie 1). As expected, actin is enriched at both competing fronts, while MRLC strongly localizes at the original cell rear. Accumulation of myosin at the losing front follows after the decision has been made^27^ (Fig. 1C). Labeling the nucleus with Hoechst and the microtubules with SiR-tubulin, we saw that in most events the nucleus and the microtubule organizing center are localized behind the obstacle apex at the moment of retraction, a behavior that is quite distinct from that exhibited by dendritic cells inside sharp Y-shaped obstacles^21^ (Fig. 1D, Supplemental Movie 2). Visual inspection revealed no obvious pre-existing asymmetries in cell shape, nucleus positioning, or in the spatial distributions of actin, myosin or microtubules that could reliably predict the turning direction.

### Faced with a symmetric dilemma, cell asymmetries arise only late in the decision-making process

**Figure 2:**
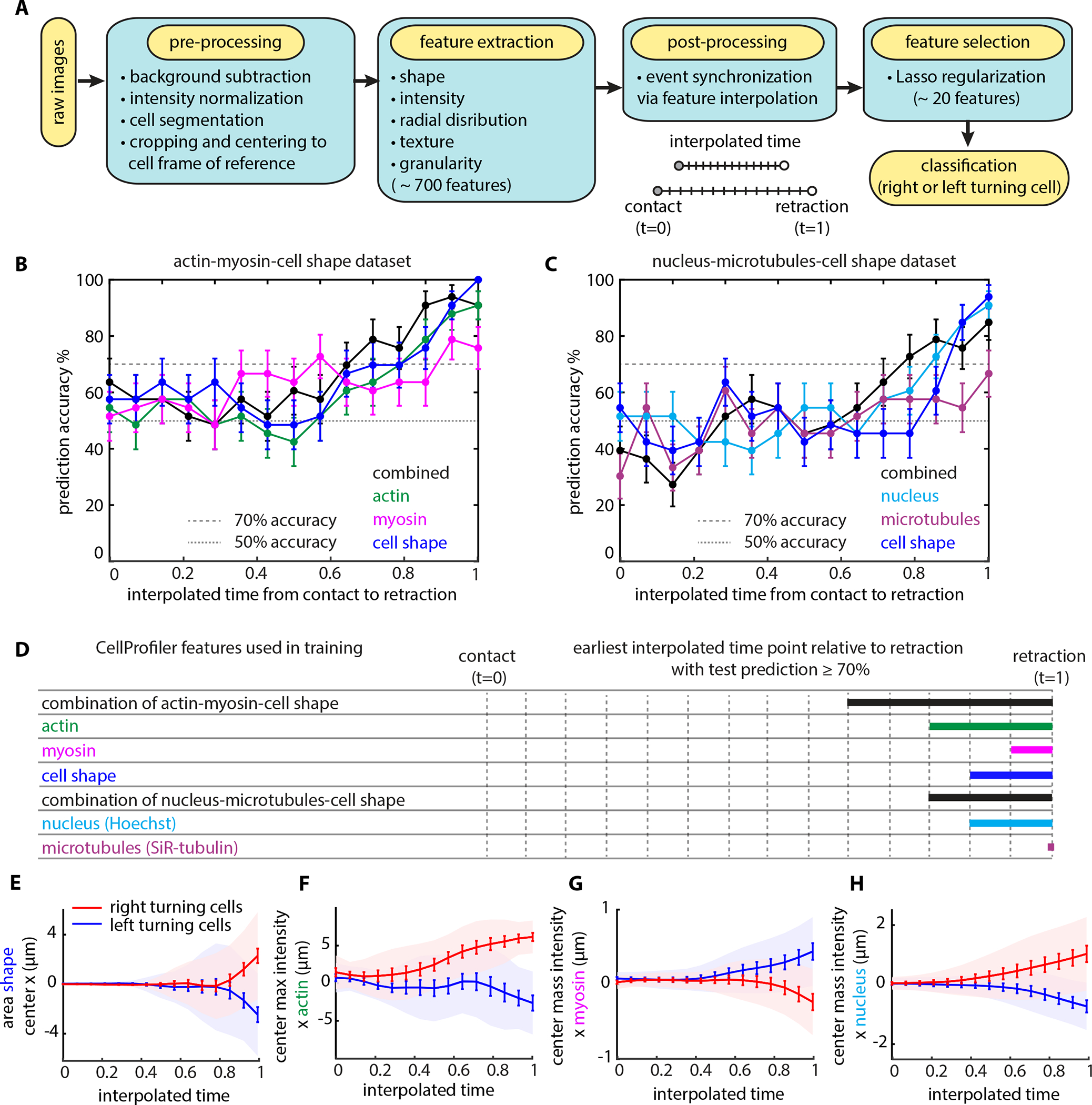
Faced with a symmetric dilemma, cell asymmetries arise only late in the decision-making process **(A)** Flow diagram of the statistical learning pipeline designed to determine whether small asymmetries in cellular structure bias or predict the turning direction of HL60 cells during symmetric decision-making. **(B-C)** Test set prediction accuracy of statistical models leveraging information contained in binary masks and fluorescent images of actin and myosin **(B)** and in binary images and fluorescent images of the cell nucleus and microtubules **(C)** across interpolated time points between contact with the obstacle and retraction initiation. **(D)** Graphic representation of the earliest time point, relative to retraction initiation, with test prediction accuracy ≥ 70% for each statistical model **(E-H)** Representative features with the most significant lasso predictive capacity; cell center *x*-coordinate **(E)**, maximum intensity of actin **(F)**, center of mass intensity (*i.e.,* intensity weighted centroid) of myosin **(G)** and of cell nucleus **(H)** over interpolated time between contact with the obstacle and retraction initiation.

To determine whether small asymmetries in cellular structure might bias or predict the decision-making, and (if so) when during the process these asymmetries might arise, we designed an agnostic statistical learning approach. Briefly, we collected time-lapse fluorescence microscopy movies of hundreds of decision-making events from single cells, capturing the organization and dynamics of different cytoskeletal components as well as cell shape-related information (Fig. 2A). We collected two datasets: one dataset composed of phase contrast images, along with fluorescence images of actin-YFP and MRLC-mApple, and another dataset composed of phase contrast images together with fluorescence images of the cell nucleus (Hoechst) and microtubules (SiR-tubulin). For both datasets, we collected over 160 events from single cells. For each event, we pre-processed the raw images and then proceeded to extract hundreds of features for each image using the open-source software CellProfiler^28,29^. These features report on shape and fluorescence intensity distribution metrics, as well as image texture and granularity.

We found that different cells resolve the competing fronts at different rates (Supplemental Fig. 1A), with decision-making time showing a negative correlation with migration speed before contact with the obstacle (Supplemental Fig. 1B). That is, cells that were moving quickly before contact with the obstacle also typically made their decision faster than did slower-moving cells. To facilitate comparisons across the large population of individual cells, we filtered the data to include only events whose time from contact with the obstacle until retraction initiation was within one standard deviation of the population average. This resulted in a final dataset size with 100 events in each dataset. To synchronize the filtered decision-making events, we worked on a rescaled time, interpolating feature values for a fixed number of equally spaced time points between contact with the obstacle and retraction initiation (Fig. 2A; see Methods).

For each dataset, out of the 100 decision-making events we randomly selected 67 for training and held back the remaining 33 as an independent test set. Logistic regression with “least absolute shrinkage and selection operator” (lasso) regularization was enforced to prevent overfitting the data^30^. Briefly, lasso regularization applies a penalty to the feature magnitudes to enforce use of only a limited number of nonzero features, leading to a sparse solution (see Methods). Leveraging lasso, we kept ~ 10 features for training our binary (left/right) classification (Fig. 2A).

Plotting the prediction accuracy for the independent test set for each dataset, we see that at the time the cell contacts the obstacle (t=0) no statistical learning model predicts the cells’ ultimate direction with accuracy better than 50% (random) (Figs. 2B–2C). We also separately interrogated the cell shape, actin, myosin, nucleus and microtubule features, but found that models constructed on individual feature sets have poorer predictive power than models constructed by combining features from different cell structures (Figs. 2B–2D). Overall, the statistical model that we derived from the combination of cell shape, actin, and myosin features was the most predictive of the cell’s final turning direction. However, even with that best model, we found that we can predict the cell’s turning direction with accuracy greater than 70% only during the last third of the decision-making process (typically about 15 s before the initiation of retraction).

Notably, the statistical model constructed on the microtubule features alone was the least predictive out of the ones examined. Microtubules have been shown to be indispensable for dendritic cell decision-making^21,31^. Nocodazole treatment, known to depolymerize microtubules, resulted in a prominent phenotypic change whereby dendritic cells were unable to initiate a retraction event, often resulting in cell breaking^21,31^. However, when we treated HL60 cells with nocodazole we did not observe such a retraction initiation defect nor cell fragmentation (Supplemental Figs. 1E–1F, Supplemental Movie 3). These results, together with the lack of predictive information associated with microtubule-based image features, suggests that HL60 cells resolve directional dilemmas using a mechanism that is distinct from that of dendritic cells.

The most highly scored individual features, as selected from the statistical models, also appear to diverge from symmetry (y=0) late in the decision-making process (Figs. 2E–2H). Different regularization methods, *e.g.,* elastic net^32^ or sparse group lasso^33^ (see Methods), did not yield models of improved prediction accuracy (Supplemental Figs. 1C–1D).

Our statistical analysis suggests that any pre-existing cell-intrinsic asymmetries, if they exist, are not reflected in the structural organization of the cytoskeleton (actin, myosin, microtubules) or the positioning of the nucleus until late in the decision-making process. This conclusion is also consistent with our earlier finding that cells can detect asymmetries in channels at least 4 μm past the obstacle apex just as efficiently as asymmetries proximal to the apex (Fig. 2B). Overall, we conclude that neutrophils resolve symmetric directional dilemmas by random choice late in the decision-making process, and we find no evidence that pre-existing asymmetries are amplified to help the cells resolve competing fronts.

### Optogenetic GPCR activation can only bias decision-making late in the process

**Figure 3:**
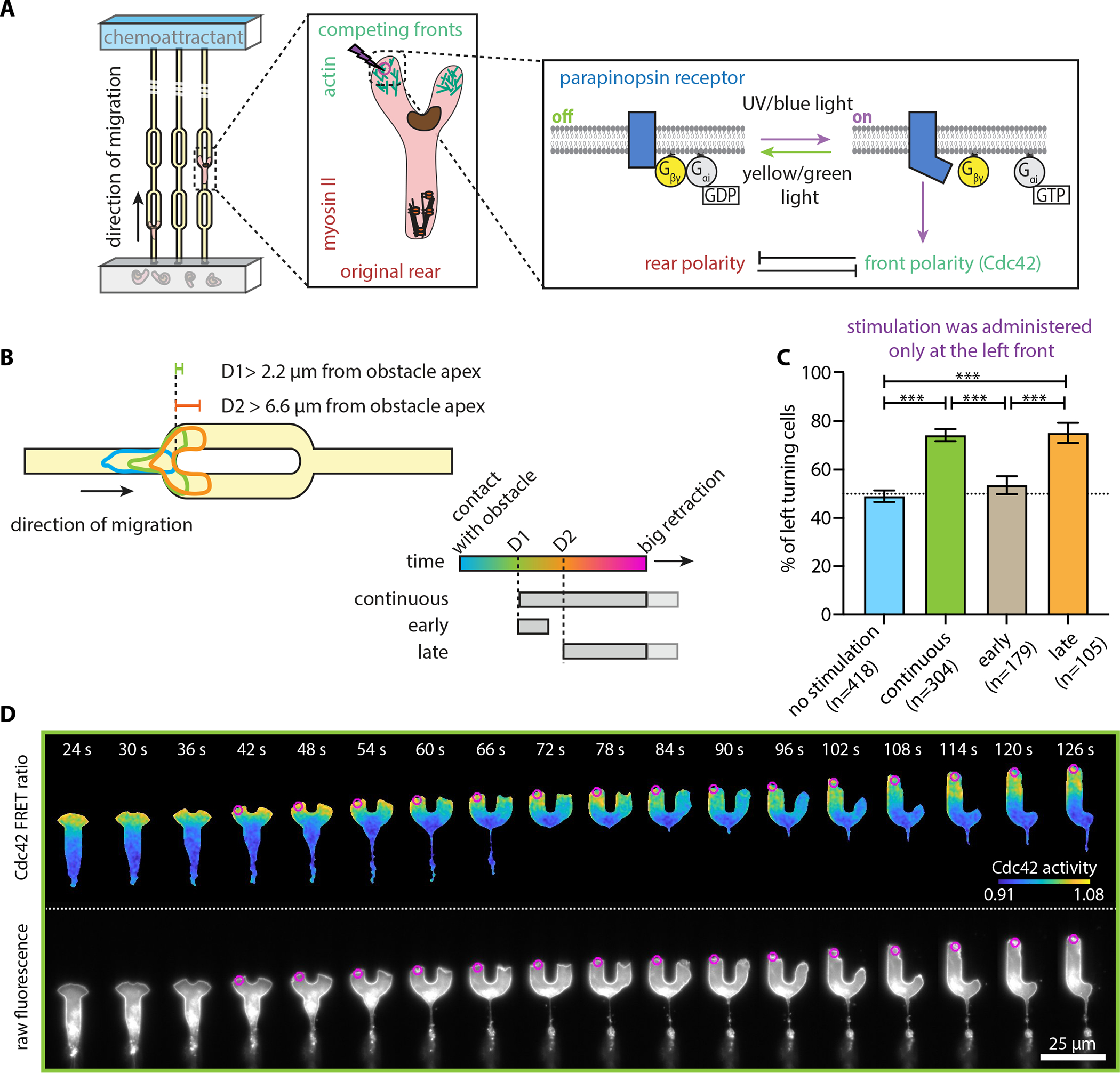
Optogenetic GPCR activation can only bias decision-making late in the process **(A)** Schematic representation of optogenetic GPCR activation during the competition. HL60 cells expressing parapinopsin, an optogenetic receptor, migrate inside the symmetrically bifurcating microfluidic channels. The opsin is turned on using UV/blue light and off with yellow/green light. Blue light stimulation (magenta circle/lightning bolt) at the left competing cell front activates the receptor. The receptor is coupled to the polarity signal transduction network and activation reinforces the front polarity module. **(B)** Schematic of the competition stimulation assays. Stimulation is initiated after the two competing fronts have clearly separated from one another (*i.e.,* when both have extended at least 2.2 μm past the apex) for the continuous and early assays; late stimulation assay starts when both sides have extended at least 6.6 μm past the apex. Continuous and late stimulation entails persistent pulsated stimulation, whereas early stimulation comprises 5 pulses. **(C)** Bar plots of the percentage of left turning cells under no stimulation (n = 418 cells), continuous (n = 304 cells), early (n = 179 cells) and late (n = 105 cells) stimulation. Error bars indicate standard deviations assuming a binomial distribution around the cumulative mean of each assay. Two-sided Fisher exact tests revealed significant differences across no stimulation and continuous and late stimulation assays (*** p < 0.001). **(D)** Live-cell imaging snapshots of a cell expressing parapinopsin and a red/far-red Cdc42 FRET sensor, representative of 304 individual cells. Cells migrate without experimental perturbation prior to initiation of continuous stimulation (magenta circles) at the left competing front (Supplemental Movie 4). Upper and lower panels show the registered sensors (gray scale) and the computed Cdc42 sensor FRET ratio, respectively. Images were captured every 3 s and subsampled for illustration purposes. Scale bar: 25 μm.

Since cells challenged with a symmetric decision-making task appear to decide late into the process, we wondered whether receptor inputs could create early asymmetry to bias and speed up decisions. We used an HL60 cell line stably expressing parapinopsin (a light-sensitive G_i_α-family GPCR) and a spectrally compatible tdTomato/tdKatushka2 FRET biosensor for Cdc42 activity^34^, a critical signaling component at the cell front^7,35–37^. This system allows direct measurements of signaling responses in cells whose migration is guided by parapinopsin^34^ (Fig. 3A). Cells were confined to migrate inside the same symmetrically bifurcating microfluidic channels described above. Using a 407 nm laser, we locally stimulated cells with 10 ms light pulses, and with spatial precision of about a 1 μm diameter spot, using real-time image analysis to position the activation spot automatically and dynamically at the appropriate location (see Methods).

We found that repeatedly stimulating the left cell front every 3 s (beginning when both had extended at least 2.2 μm past the apex), was sufficient to bias the turning direction (75% left turning cells vs. 49% under no stimulation) (Figs. 3B–3C, Supplemental Movie 4). Given previous experimental results using optogenetic stimulation of this kind^34,38^, we were pleased but not surprised that this “continuous stimulation” assay was sufficient to bias the turning direction of a cell population. In addition, the continuous optogenetic GPCR activation resulted in marginally greater directional biasing as compared to the channel geometry asymmetries examined before (Fig. 1B).

However, we noticed that stimulation did not resolve the competition immediately (Fig. 3D). This observation raised the question of whether receptor inputs early in the competition create asymmetry which is maintained or amplified to affect the ultimate outcome, or whether these inputs have little effect because each cell front is strongly committed to its protrusive state. To probe this question, we devised an “early stimulation” assay, delivering 5 stimulation pulses early on during the decision-making process, with the same stimulation onset as in the continuous experiment (Supplemental Movie 5). We discovered that this early intervention (in an attempt to bias signaling before the development of overt cytoskeletal structural asymmetries) had no effect, with the direction of the final behavioral decision returning to the stochastic 50%−50% left-right baseline. This result indicates that inducing a transient signaling asymmetry at an early stage is not sufficient to drive the system out of its balanced state, either by strengthening the stimulated edge or by weakening the competing edge.

Our results thus far suggested that decision-making happens late, and we reasoned that this may also be the time when asymmetric receptor inputs have the greatest effect. To test this directly, we devised a “late stimulation” assay (Supplemental Movie 6), where we stimulated the left front only after the competing fronts had extended 3-fold further than the continuous and early stimulation assays (about 6.6 μm past the apex of the obstacle) (Figs. 3B–3C). We found that late stimulation was just as effective as the continuous stimulation, providing additional support that early stimulation has little effect and that optogenetic GPCR activation can bias decision-making only late in the process.

### Decision-making follows a stereotypical course dominated by autonomous polarity and cell stretching

**Figure 4:**
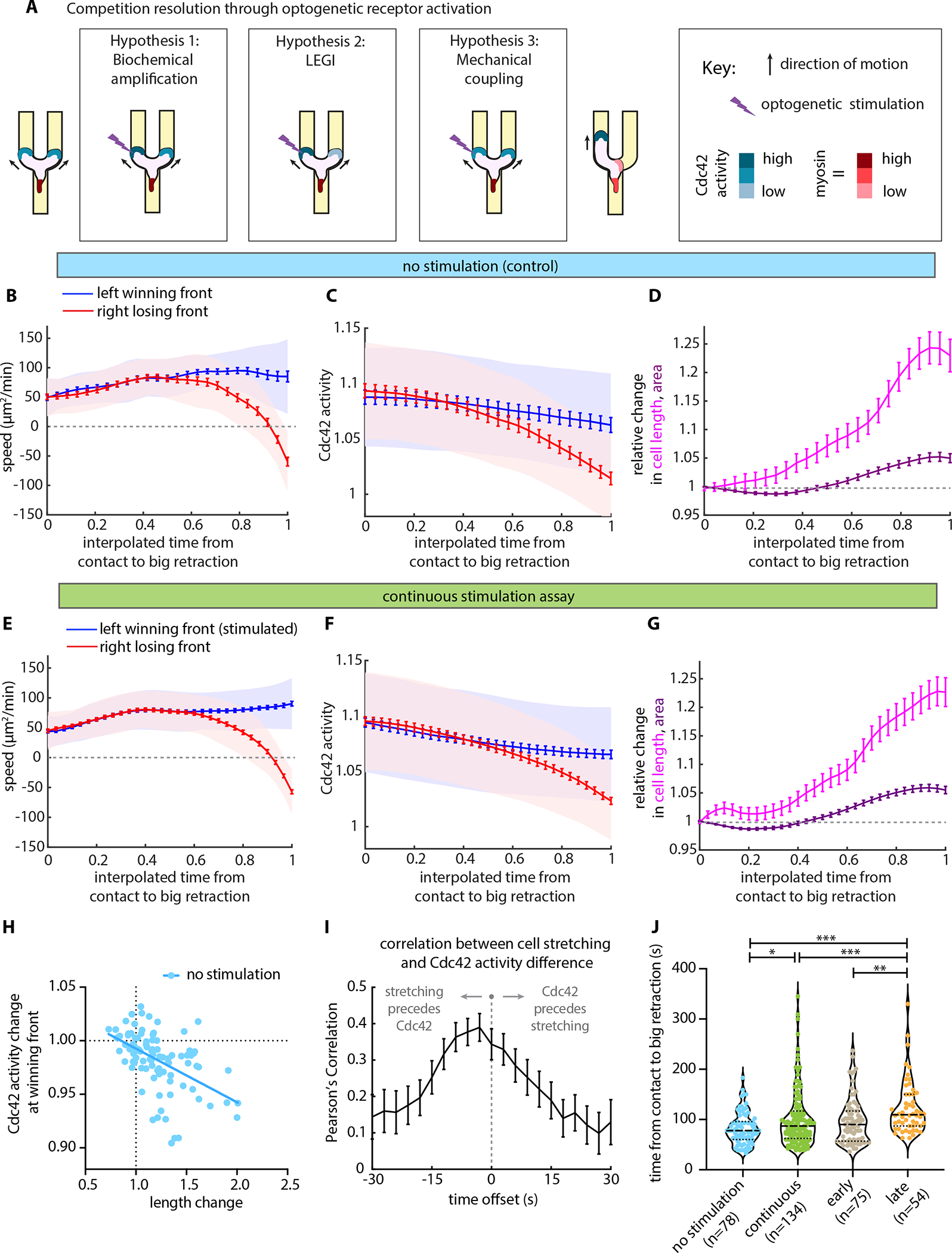
Decision-making follows a stereotypical course dominated by autonomous polarity and cell stretching **(A)** Optogenetic stimulation experiments can distinguish among models for coordination between outcomes at competing fronts. Hypothesis 1: If biochemical amplification dominates coordination between fronts, then local receptor activation on the left side would be expected to increase or maintain Cdc42 activity on that side. Hypothesis 2: Under a LEGI model, local receptor activation on the left side would be expected to cause a decrease in Cdc42 activity on the right side. Hypothesis 3: If mechanical stretching of the cell membrane or cortex limits local Cdc42 activity, then local receptor activation on the left side during competition may have no effect on Cdc42 activity on either side. Mean speed **(B)** and magnitude of the Cdc42 activity (FRET ratio) **(C)** of the winning (blue) and the losing (red) competing fronts for 48 spontaneously left turning cells that received no stimulation (lines: means, shaded regions: SD, error bars: mean value ± SE). **(D)** Relative change in cell length (magenta) and cell area (purple), both normalized to the initial length and area at the time of contact with the obstacle, for 100 unstimulated (control) cells (lines: means, error bars: mean value ± SE). Mean speed **(E)** and magnitude of Cdc42 activity **(F)** of the winning (blue) and the losing (red) competing fronts for 125 stimulated left turning cells (lines: means, shaded regions: SD, error bars: mean value ± SE). **(G)** Relative change in cell length (magenta) and cell area (purple), both normalized to the initial length and area at the time of contact with the obstacle, for 166 continuously stimulated cells (lines: means, error bars: mean value ± SE). **(H)** Scatter plot of Cdc42 activity sensor fold-change and at the winning front vs. length change of 94 unstimulated cells. Changes were calculated from when the two competing fronts had clearly separated from one another (*i.e.,* when both had extended at least 2.2 μm past the apex) and until big retraction initiation. Linear best-fit curve has been provided to guide the eye. **(I)** Temporal cross-correlation between the relative change in cell length and the absolute magnitude of the difference in Cdc42 activity between the winning and losing fronts as a function of time offset, for 75 unstimulated (control) cells (line: mean, error bars: mean value ± SE). The temporal analysis was performed for the same observation window as panel **(H)**. **(J)** Violin plots of the time from contact with the obstacle to big retraction of cells under no stimulation (n = 418 cells), continuous (n = 244 cells), early (n = 69 cells), and late (n = 97 cells) stimulation; *p*-values of two-sided Wilcoxon’s rank-sum test (*p < 0.05, **p < 0.01, ***p < 0.001). For this analysis, cells were filtered so that all cells analyzed would have initiated the late stimulation protocol, if given the chance. In other words, only cells that extended their competing fronts at least 6.6 μm past the obstacle apex.

Next, we sought to identify the underlying mechanism that allows the cells to become more amenable to directional bias late into their decision-making process. We began by determining whether changes in signaling activity precede and might determine the symmetry breaking in protrusion at the two fronts. Different hypotheses for mechanisms of coordination between the two fronts during the decision-making process predict distinct outcomes for the changes in Cdc42 activity following optogenetic stimulation of one side (Fig. 4A).

As a control, we quantified the mean protrusion speed and magnitude of the Cdc42 activity of the two competing fronts for left-turning unstimulated control cells (Figs. 4B–4C). Examining the first half of the decision-making process (from 0 to 0.5 in interpolated time), we could detect no obvious difference either in speed or in Cdc42 activity between the two competing fronts. These observations are reminiscent of our analysis described above, where cytoskeletal asymmetries could be detected only very late in the decision-making process. About 60% of the way through the process, both the speed and the Cdc42 activity of the two competing fronts began to diverge. Nevertheless, the losing front continued to move forward, just at a slower speed than the winning front, until the forward progress of the losing side stalled and began to retract about 90% of the way through the decision-making process (Fig. 4B). The deviation of Cdc42 signaling between the two competing fronts was similarly subtle and gradual (Fig. 4C). Notably, during this competition phase, the absolute amount of Cdc42 activity gradually decreased for both the left and right sides, but the rate of the activity decreased slightly more rapidly for the losing side (Fig. 4C). In addition, we observed gradual stretching of the cells during their decision-making (Fig. 4D).

Comparing left-turning cells whose left front had been optogenetically stimulated (Figs. 4E–4G) with control cells that spontaneously turned left with no stimulation (Figs. 4B–4D), we found no measurable differences in cell stretching, protrusion speed, or the magnitude of the Cdc42 activity at either the winning or the losing front. These results are consistent with a model in which a strong self-organizing property of the polarity machinery drives the behavioral response. Therefore, even though continuous (and late) optogenetic GPCR activation clearly influences the turning direction across the population of cells, our results suggest that the autonomous polarity program dominates at the level of signaling in single cells, and that parapinopsin activation is only biasing the polarity machinery instead of completely overwriting it.

Even so, the fact that Cdc42 activity gradually declines at both fronts during the competition suggests that front polarity may be weakening. We hypothesized that mechanical cell stretching could be causing this weakening and thereby mediating the competition. To test this, we analyzed stretching and signaling at the single cell level. We found that the degree of cell stretching correlates with the drop in Cdc42 activity (Fig. 4H). Notably, cells that did not have apparent stretching showed very little change in Cdc42 activity, but cells that stretched to larger degrees tended to have correlated decreases in front signaling activity (Fig. 4H). Furthermore, cross-correlation analysis indicates that stretching is temporally correlated with and slightly precedes the divergence in Cdc42 signaling between the two fronts (Fig. 4I). Our results are consistent with previous findings that aspiration-mediated increase in mechanical tension in neutrophil-like cells is sufficient to cause long-range inhibition of actin assembly and Rac activity at the cell front^25,26^, and they suggest that stretching may mediate competition by weakening both fronts and making them more amenable to external biasing.

Finally, when comparing the duration of the decision-making process, measured from contact with the obstacle to onset of big retraction (projected retraction area larger than 7.2 μm^2^), continuous and late stimulation assays show longer time averages, with more decision-making events requiring extended time to resolve, as compared to spontaneous resolution of the dilemma in unstimulated cells (Fig. 4J). That is, stimulation has a clear measurable effect on the system, but only by prolonging the decision-making process and biasing the ultimate outcome.

### A committed retracting edge is unresponsive to receptor input until it is fully retracted

**Figure 5:**
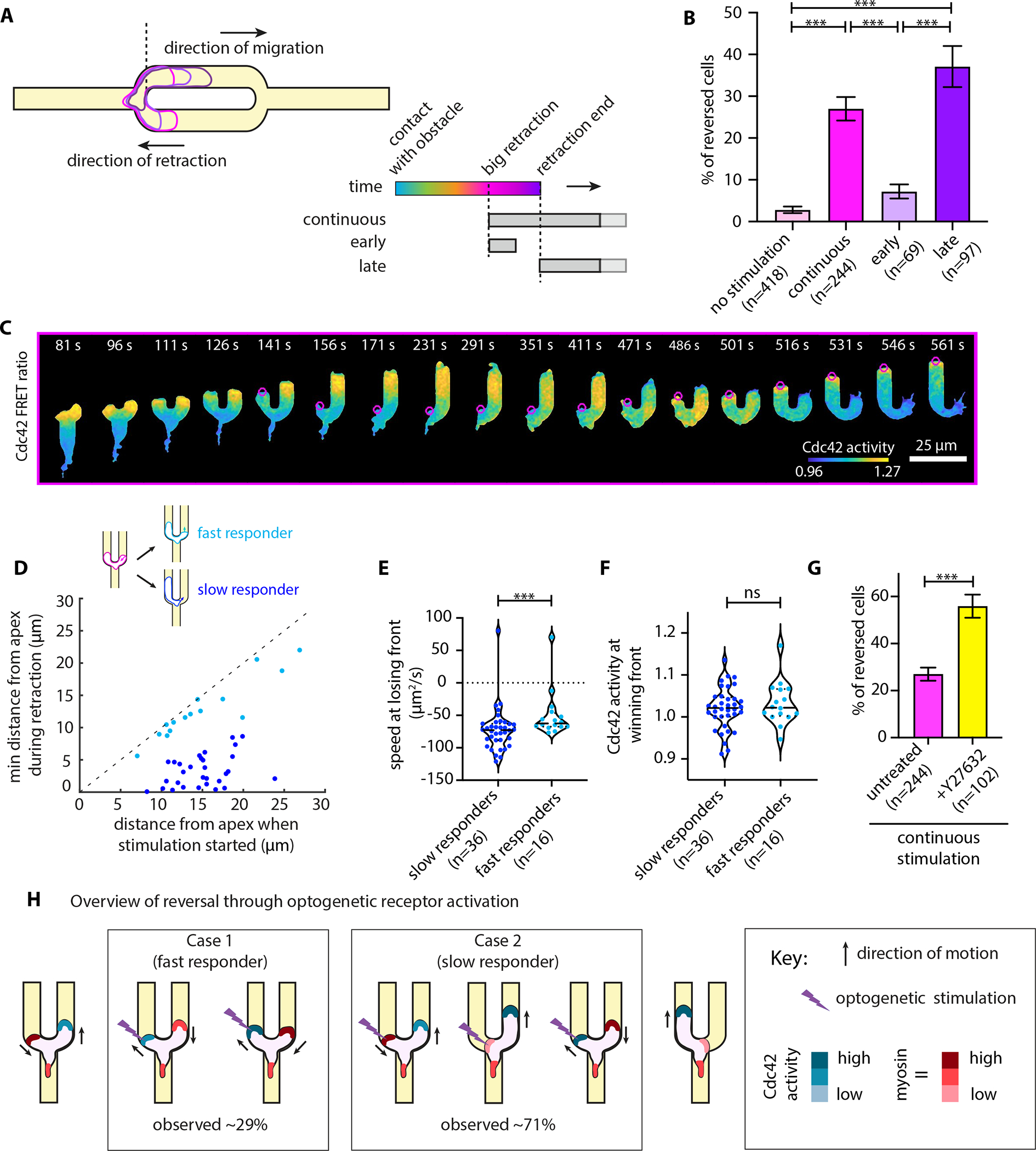
A committed retracting edge is unresponsive to receptor input until it is fully retracted **(A)** Schematic illustration of the reversal stimulation assays. Stimulation initiation commences after we detect a big retraction of a competing front (*i.e.,* retraction > 7.2 μm^2^) for the continuous and early assays; late stimulation assay commences when the retracting edge has reached the apex midline. Continuous and late stimulation entails persistent pulsated stimulation, whereas early stimulation comprises 5 pulses. **(B)** Bar plots showing the percentage of reversing cells under no stimulation (n = 418 cells), continuous (n = 244 cells), early (n = 69 cells) and late (n = 97 cells) stimulation. Error bars indicate standard deviations assuming a binomial distribution around the cumulative mean of each assay. Two-sided Fisher exact tests revealed significant differences across no stimulation and continuous and late stimulation assays (*** p < 0.001). **(C)** Live-cell imaging snapshots of a cell expressing parapinopsin and a red/far-red Cdc42 FRET sensor, representative of 244 individual cells. Cells migrate and decide on a direction of turning without experimental perturbation. Upon identifying a big retraction, continuous stimulation (magenta circles) at the retracting/losing front commences (Supplemental Movie 7). Panels show the computed Cdc42 sensor activity. Images were captured every 3 s and subsampled for illustration purposes. Scale bar: 25 μm. **(D)** Scatter plot of minimum distance from the obstacle apex during retraction of the losing front vs. distance of the same front from the obstacle apex when the stimulation started, for 45 reversing cells. Cells stratified as slow (blue) and fast (cyan) responders. **(E-F)** Violin plots of speed of the losing/retracting front **(E)** and Cdc42 activity at the winning front **(F)** at the time of big retraction initiation for slow (n = 36 cells) and fast responders (n = 16 cells) under the continuous reversal assay; *p*-values of two-sided Wilcoxon’s rank-sum test (*** *p* < 0.001, ns: *p* > 0.05). **(G)** Bar plots of the percentage of reversing cells under no treatment (n = 244 cells) and Y2763 treatment (n = 102 cells). Error bars indicate standard deviations assuming a binomial distribution around the cumulative mean of each condition Two-sided Fisher exact tests revealed a significant difference between conditions (*** p < 0.001). **(H)** For reversal experiments, we observed that the minority of the cells (29%) showed an immediate rise of Cdc42 activity at the stimulated cell edge, accompanied by protrusion of the cell edge in the direction of stimulation (Case 1). For most of the cells (~71%) we observed that the retracting edge continued to retract all the way back to the cell body before it began to extend again in the direction of stimulation (Case 2).

Finally, we asked whether we could rescue a losing front and reprogram it to start protruding again. To that end, we let cells spontaneously pick a direction, but once we detected a big retraction (projected retraction area larger than 7.2 μm^2^), we started repetitively stimulating the retracting side. We called this assay “continuous stimulation” (Fig. 5A, Supplemental Movie 7). Similar to the competition assays described above, we also devised an “early stimulation” assay, where we stimulated the retracting edge with only 5 pulses (Supplemental Movie 8), as well as a “late stimulation” assay, where we let the cell retract all the way back to the apex of the obstacle and then began repetitively stimulating the previously retracting side (Fig. 5A, Supplemental Movie 9). We found that continuous stimulation was sufficient to reverse 27% of cells (Fig. 5B). Early stimulation was much less effective, only reversing 7% of cells, whereas late stimulation was even more effective than continuous stimulation, reversing 37% of cells (Fig. 5B).

Even for the cells that were amenable to optogenetic reversal, we found that the response did not occur immediately. Specifically, we found that 71% of reversing cells, despite our continuous stimulation, kept retracting their losing front all the way back to the obstacle apex before they started reversing (Figs. 5C–5D). For these slow-responding cells, the losing front appears to enter a refractory period that requires complete retraction to the cell body before stimulation can reorient them. By classifying cells into slow and fast responders, we found that the slow responders typically had a faster retracting speed at the start of stimulation (Fig. 5E). We found no difference in the magnitude of Cdc42 activity at the winning front between slow and fast responders (Fig. 5F). That is, it appears that a losing front with high retraction speed tends to keep retracting, and only after it has completed its retraction program will it become amenable to new receptor inputs and switch into a protrusive behavior, ultimately leading to cell reversal.

Prior work has shown that myosin II contractile activity at the cell’s rear contributes to the maintenance of polarity, while also inhibiting cell front polarity programs^11,38–40^. We therefore sought to test the effects of altering myosin contractility in our optogenetic reversal assay. We found that ROCK inhibition with Y27632 treatment, known to decrease MRLC phosphorylation and myosin II activity in neutrophils^27,41^, increases the percentage of reversing cells (Fig. 5G). Similar to what we previously found for optogenetic receptor-directed cell reversals in 1-D straight channels^38^, this suggests that the refractory nature of the retracting edge is at least in part mediated by local myosin II/RhoA activity.

Notably, and consistent with our previous observations in straight channels^38^, the direction of migration reverses considerably before the two cell edges reach equal levels of Cdc42 activity and myosin II localization (cross-point) (Supplemental Figs. 2A–2D). That is, the cell edge that has more Cdc42 activity at any particular moment in time is not necessarily the driving front. Comparing untreated cells to Y27632-treated cells, the latter exhibit faster Cdc42 and myosin dynamics, with the Cdc42 and myosin cross-points happening earlier than in untreated cells but still trailing behind the directional reversal (Supplemental Figs. 2E–2H).

Taken together, our results suggest that, once a cell has made a directional choice, the losing front may enter a refractory period that requires complete retraction before receptor activation can reprogram the edge to switch back to a protrusion program (Fig. 5H). The refractory nature of the retracting edge can be dampened by reducing myosin II activity.

## DISCUSSION

**Figure 6:**
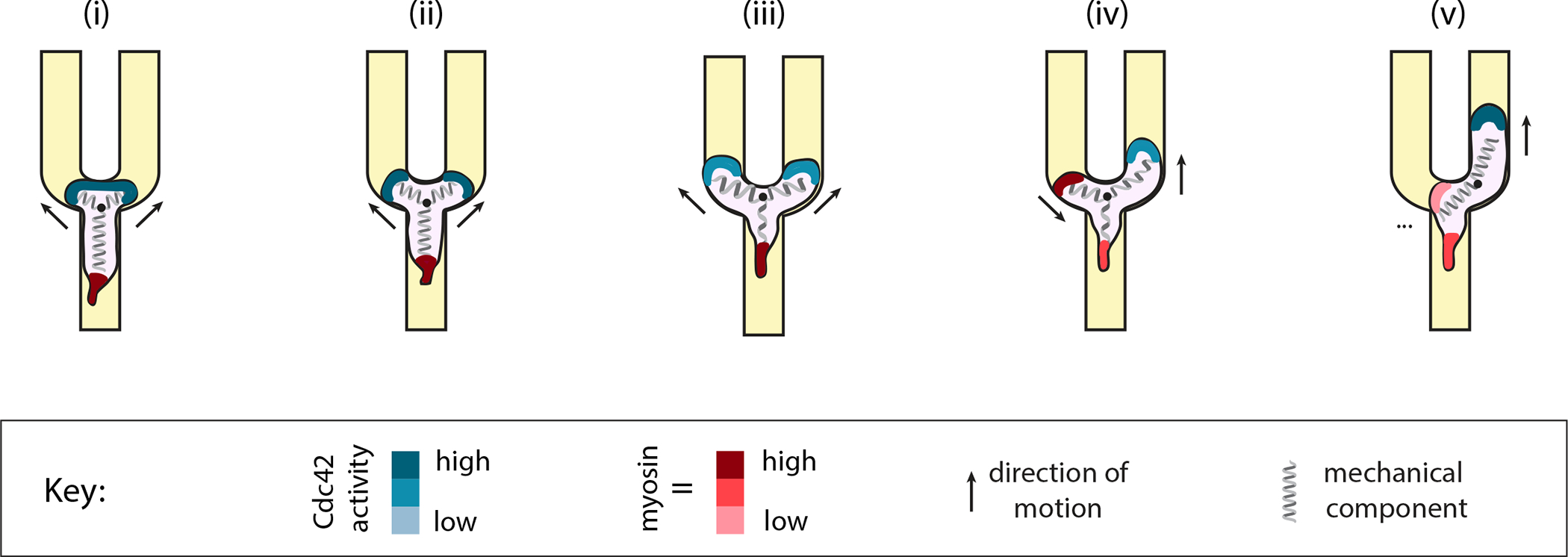
Conceptual model of decision-making in a symmetric directional dilemma A polarized cell encounters a bifurcation with high Cdc42 activity at the front and high myosin II at the rear (i). After the leading edge is forced to split into two (ii), the activity of Cdc42 decreases in both competing fronts as they continue to protrude into the two channels and the mechanical elements of the cell become stretched (iii). Ultimately, one of the two competing fronts “wins” and myosin II accumulates in the newly retracting “losing” side (iv). After the dilemma is resolved, the cell re-establishes a simple polarization state with high Cdc42 at the front and high myosin II at the rear, stably migrating upward in only one of the two bifurcating channels (v).

Overall, our results indicate that cells respond to receptor inputs in a context-dependent way; that is, cell edges locally committed to protrusion or retraction programs can be insensitive to receptor inputs, and coordination between competing fronts is primarily mediated by mechanical stretching (Fig. 6). At the beginning of the competition, the branched cell morphology is a stable state with two equally viable directional options, because both leading edges are committed fronts reinforced by positive feedback. As the cell extends further, mechanical stretching of the cell membrane and cortex limits the continued growth of the actin network at the two leading edges, and causes a decrease in Cdc42 activity. The weakening of the “frontness” signal at both competing leading edges renders the system more sensitive to noise and receptor inputs, which promotes a resolution. Consistent with this interpretation, our statistical learning methods were unable to detect predictive cell asymmetries until late in the process, and our optogenetic experiments indicated that the fronts were only responsive to receptor inputs during this same time window. Finally, after retraction begins, a retracting edge becomes committed to full retraction and typically retracts all the way back to the cell body before it becomes receptive to new receptor inputs.

An intriguing observation of this work is that both the protrusion speed and the level of Cdc42 activity at a leading front are largely the same with or without optogenetic receptor stimulation (Fig. 4). While the polarity circuit is known to have self-organizing characteristics^5,42^, this result was still surprising, given the connections from receptors to front signaling components. However, previous analysis of neutrophil-like cells migrating under agarose found that the front-to-back spatial profiles of Rho GTPases appeared almost indistinguishable irrespective of whether a cell was migrating randomly or in a gradient^7^. These findings suggest that the cell-intrinsic polarity circuit largely defines the pattern of Cdc42 activity and the other GTPases. Receptor input stimulates and directs the system, but as the system adapts to gradients and inputs, it eventually settles into a more cell-autonomous pattern. Hence, early during the competition the two fronts are fully committed, and the front machinery is maximally engaged. Our optogenetic stimulation does shift the population average behavior, but that is hard to detect at the level of a specific signaling activity like that of Cdc42.

Our data are most consistent with a simple model where a mechanical integrator, cell stretching, contributes to the decline of Cdc42 activity (Fig. 4). The downward trend of the Cdc42 activity at both fronts suggests that cell stretching is impacting the strength of polarity at both fronts, not just at the eventual losing front. Our observations contradict the predictions of a LEGI model, where we would expect to see a decrease in Cdc42 activity at the losing front under optogenetic stimulation, as compared to the activity in the losing front of unstimulated cells undergoing a spontaneous directional choice. However, we were unable to detect any such difference. Instead, the Cdc42 activity measurements shown in Fig. 4 are most consistent with a simple mechanical coupling between the two cell fronts, without the need to invoke an additional LEGI-type chemical signal that communicates the status of one front to its competitor. One appealing candidate for a mechanical integrator that could act in this way is the in-plane tension in the plasma membrane or cell cortex^25,26^. In rapidly-moving fish epidermal keratocytes, membrane tension is largely determined by cytoskeletal forces^43^ and is concomitantly highest at the protruding leading edge^44^. In HL60 cells, increasing membrane tension is sufficient to suppress actin polymerization, and also interferes with recruitment of the SCAR/WAVE complex (an upstream regulator of actin polymerization) at the leading edge^25^. The closed geometry of our microfluidic channel system makes it difficult to locally alter membrane tension in one of the two leading edges of a cell forced to bifurcate, but we would predict that a local perturbation in membrane tension should provide a stronger directional bias than we have observed for local optogenetic receptor activation.

From a functional perspective, the context-dependent response to receptor inputs and the strong commitment to local protrusion and retraction programs that our work revealed could represent a particularly efficient way for cells to navigate noisy and physically complex environments during immune surveillance. When cell movement is unimpeded, the membrane tension should remain at a steady state, allowing the protruding front and retracting rear domains to persist for efficient migration. However, tension will inevitably increase when the cell is stuck or conflicted with two or more competing leading edges pulling in different directions. The global increase in tension then renders all the competing leading edges more sensitive to noise and to new receptor inputs, promoting resolution of the conflict. One consequence of this mechanical sensitization mechanism is that cells might not always end up moving in the direction with the strongest signal or steepest chemical gradient. Indeed, in cases where the chemical gradient alone might lead cells into an intractable or overly restrictive mechanical path, it is critical that cells have a mechanism to resolve these conflicts without fragmenting or becoming trapped. In these cases, the commitment of a cell front moving in a locally suboptimal direction could provide a solution, allowing the cell to escape and maintain persistent migration. For ultimately reaching their target, this persistence would be more important than short-term accuracy, as the cell can always correct its course later. That is, for an immune cell, it may be better to run now and figure out the details later, rather than to sit in place fixated on the most direct path.

## STAR METHODS

### RESOURCE AVAILABILITY

#### Lead contact

Further information and requests for reagents may be directed to and will be fulfilled by the Lead Contact Julie Theriot jtheriot@uw.edu (J.A.T.).

#### Materials availability

Materials developed in this study are available on request to the corresponding authors.

#### Data and code availability

- The data that support the findings of this study are available from the corresponding authors upon request.
- The code we developed for automating experiments and data analysis is available from the corresponding authors upon request and is also publicly available at: https://gitlab.com/theriot_lab/neutrophil-decision-making (DOI: 10.5281/zenodo.7535222).
- Any additional information required to reanalyze the data reported in this paper is available from the lead contact upon request.

### EXPERIMENTAL MODEL AND SUBJECT DETAILS

#### Cell culture and differentiation

The parental HL60 cell line was a generous gift from Orion Weiner’s lab. In this work, we used the previously published HL60 cell line expressing actin-YFP and MRLC-mApple^27^, an HL60 cell line expressing the tdTomato/tdKatushka2 FRET biosensor for Cdc42 and the light-controllable parapinopsin GPCR^38^, and an HL60 cell line expressing mScarlet-I-tagged Myl9 and parapinospin^38^. Cells were cultured and differentiated into a neutrophil-like state as previously described^46^. Briefly, cells were cultured in complete RPMI media (RPMI 1640 plus L-glutamine and 25 mM HEPES media (Gibco RPMI 1640 Medium, HEPES, Thermo Fisher Scientific), supplemented with 10% heat-inactivated fetal bovine serum (hiFBS) (Foundation Fetal Bovine Serum, Gemini Bio, heated in a water bath for 40 min at 56 °C), 100U/mL penicillin, 10 μg/mL streptomycin, and 0.25 μg/mL Amphotericin B (Gibco Antibiotic-Antimycotic, Thermo Fisher Scientific)). Cells were maintained at 37 °C and 5% CO2 in a tissue culture incubator and were passaged every day to maintain a cell density close to 5×10^5^ cells/mL. HL60 differentiation into a neutrophil-like phenotype was achieved by diluting cells in complete RPMI media containing 1.3% DMSO (Acros 61097) at a starting density of 2×10^5^ cells/mL. For all the experiments, only cells differentiated for 6 or 7 days were used.

### METHOD DETAILS

#### Microfluidic device fabrication

Polydimethylsiloxane (PDMS) microfluidic devices were fabricated using standard photolithography and soft lithography approaches. Briefly, we designed two photolithography masks using AutoCAD (Autodesk), one mask for the cell loading channels and dead-end chemoattractant reservoirs, and one mask for the migration channels integrating the bifurcation challenges. Masks were prepared as high-resolution chrome on glass by Front Range Photomasks (Lake Havasu City, Arizona).

To pattern the silicon wafer master-mold, we performed two cycles of spin-coating negative photoresist (SU-8, Microchem, Newton, MA) and exposure to ultraviolet (UV) light though the appropriate masks to produce the migration channels at 3 μm height and cell-loading channels and chemoattractant reservoirs at 200 μm height.

PDMS devices were prepared using the patterned wafer as a replica mold. We mixed PDMS base (Fisher Scientific, Fair Lawn, NJ) with the curing agent at a ratio of 10:1, poured over the wafer, and degassed for at least 1 hour. After baking overnight at 75 °C, PDMS was allowed to cool, cut from the wafer, and inlets and outlets punched using the 1 mm biopsy punch (Harris Uni-Core, Ted Pella, Inc., Redding, CA). Devices were treated with oxygen plasma and bonded to glass-bottom 35 mm γ-irradiated culture dishes with No. 0 coverslips (MatTek Corp., Ashland, MA) by heating to 85 °C for 10 mins on a hotplate.

#### Microfluidic device priming

Priming of each device commenced by pipetting 20 μL of an fMLF-based chemoattractant solution through the loading port. For this, fMLF (Sigma Aldrich, stored in stock solutions of 1 μM in DMSO) was diluted in L-15 media (Thermo Fisher Scientific) containing 20% hiFBS to a final concentration of 100 nM. The device was then placed in a vacuum desiccator connected to house vacuum (27 inHg) for 10 min. Upon removal the device was rested for an additional 10 min, until the chemoattractant filled entirely the straight migration channels, connected orthogonally to the central loading channel. The device was then washed twice, by pipetting 200 μL of 10 μg/mL fibronectin (Sigma Aldrich) diluted in L-15 media containing 20% hiFBS into the loading port. These washing steps removed the chemoattractant from the central loading channel and its passive diffusion from the migration channels into the central loading channel established a chemoattractant gradient. To prevent evaporation, 3 mL of L-15 media were added to the glass-bottomed dish to cover the device.

#### Cell staining

To fluorescently label the cell nucleus and microtubules (Figs. 1–2), 3×10^5^ cells were spun down at 200 g for 5 min and resuspended in 1 mL L-15 media containing 1μL SiR-tubulin (Cytoskeleton Inc.) from a stock solution of 1 mM. The stained cell suspension was incubated at 37°C for 45 min. For the last 15 min Hoechst 33324 was added to the cell suspension at a final concentration of 1 μg/mL. Stained cells were spun down at 200 *g* for 5 min and were resuspended in ~20 μl of L-15. Out of this cell suspension, 10 μL were pipetted into the loading port of the device. The device was then transferred at the microscope stage, for subsequent cell imaging. The microscope was maintained at 37 °C for at least 30 min prior to imaging.

#### Retinal preparation and incubation

Preparation of retinal stock solutions was performed in a dark room with red-light sources^34^. Briefly, 9-cis-Retinal (Sigma Aldrich R5754) was dissolved in argon-purged 200 proof ethanol (Sigma Aldrich) to reach a concentration of 10 mg/mL. Aliquots were stored at −80 °C in small amber glass tubes (Sigma Aldrich). 0.1 g of Bovine Serum Albumin (BSA), Fraction V—Low-Endotoxin Grade (Gemini Bio 700–102P) was mixed in 10 mL of L-15 media, to prepare a 1% BSA solution. In darkness, 10 μL of retinal stock was diluted to a working concentration of 10 μg/mL by gradually adding the 1% BSA solution until all 10 mL were used. The final retinal solution was mixed overnight at 4 °C on a rocker in total darkness. Final retinal solutions were used for experiments within 2 days after preparation.

For the stimulation assays, 3 × 10^5^ differentiated HL60 cells were spun down at 200 g for 5 min and re-suspended in 1 mL of retinal solution to incubate at 37 °C for 1 h. This incubation and all remaining cell handling happened in darkness with red-light sources. Incubated cells were spun down at 200 g for 10 min and were resuspended in ~ 20 μL of L-15. Out of this cell suspension, 10 μL were pipetted into the loading port of the device. The device was then transferred on the microscope at 37 °C, where the cells would be imaged.

#### Pharmacological treatments

To explore the role of microtubules in decision-making (Supplemental Fig. 1) as well as the role of myosin II/RhoA in creating the refractory nature of the retracting losing front to receptor inputs (Fig. 5 and Supplemental Fig. 2) we pre-treated cells with either 50 μM nocodazole (Sigma Aldrich) or 10 μM Υ−27632 (Sigma Aldrich) for the last 30 min of the staining or retinal incubation, respectively. Cells were spun down at 200 g for 5 min and were re-suspended in ~ 20 μL of L-15 containing the same final drug concentration. Of this cell suspension, 10 μL were loaded in a device that was pre-treated with the same drug concentration. The pharmacological compound was included in both the chemoattractant solution as well as the washing solution used during device priming, in the same final concentration. For all pharmacological treatments DMSO was used as a vehicle. We performed control experiments with 0.2% DMSO to match the maximum final DMSO concentration in the pharmacological treatments. We found no significant differences between untreated and DMSO-treated cells.

#### Fluorescence microscopy for phase contrast and intracellular structures

Multi-channel time-lapse sequences of epifluorescence (to image the fluorescently tagged cytoskeletal structures) and phase contrast images (to image the cell shape) were acquired using an inverted Nikon Eclipse Ti2 with an EMCCD camera, Andor iXon3 (Andor Technologies). A Lambda XL (Sutter Instruments) light source was used for epifluorescence illumination. A filter wheel in the light source permits rapid selection of excitation wavelength. Images were captured every 3 s using either a 20× 0.75 NA Plan Apo air objective (for Fig. 1B) or a 100× 1.45 NA oil immersion objective (for Figs. 1C–1D, Fig. 2 and Supplemental Fig. 1). An acrylic microscope incubation chamber (Haisen Tech) was used to maintain the microscope at 37 °C during the entire imaging session. Cells were imaged using sequential phase contrast and epifluorescence illumination. To capture single cell actin-myosin dynamics we used a dual filter cube (Chroma, #59022) to capture eGFP and mCherry emission by sequential excitation with 470/40 nm and 560/40 nm filters respectively. To record Hoechst-stained cell nucleus and SiR-tubulin-stained cell microtubules signals we used standard DAPI and Cy5 (Chroma, #49006) filter cubes, respectively. Micro-Manager was used to operate all microscopy equipment^47^.

#### Microscope configuration for Cdc42 TomKat FRET sensor and Myl9 imaging

Fluorescence microscopy for the Cdc42 TomKat FRET sensor and the Myl9 imaging was performed on a Nikon Eclipse Ti inverted microscope with a XLED1 LED (Excelitas) light source for epifluorescence illumination. Images were acquired every 3 s on two Andor Zyla 4.2 sCMOS cameras, using a Cairn TwinCam LS image splitter equipped with a 605 nm edge wavelength dichroic mirror (Chroma ZT594rdc) and emission filters to allow simultaneous imaging. The Cdc42 TomKat FRET imaging was performed as previously described^34^. For myosin Myl9 imaging experiments, we used a 405/488/561/640 nm quad band filter cube (Chroma 91032) that allowed rapid, sequential 407 nm optogenetic stimulation, and simultaneous imaging of mScarlet-I-Myl9 (~ 561 nm excitation), and cytoplasmic iRFP (~ 640 nm excitation). Bandpass emission filters were used to eliminate bleedthrough of iRFP into the myosin channel. Some bleedthrough of mScarlet signal into the iRFP channel was unpreventable and tolerated to allow simultaneous imaging. Rapid and precise stage movements were achieved using an ASI stage (MS-2000 Flat-top) equipped with linear encoders. The microscope was controlled by custom-built MATLAB R2015a software (MathWorks) interfaced with Micro-Manager (Version 1.4.23), allowing automated and highly reproducible experimental conditions for cell stimulation and time-lapse microscopy protocols. Cells were imaged with a 60x oil immersion objective (Nikon Apochromat, 1.49 NA) and were maintained at 37 °C using a temperature and humidity control unit (OkoLab Microscope Lexan Enclosure). Each experiment was terminated within 2 h of imaging.

#### Stimulation assays

Cell stimulation and imaging was performed using a custom-build MATLAB R2015a interface for Micro-Manager (Version 1.4.23). Opsin stimulation was performed using a 407 nm laser (Coherent Cube) with a custom fiber coupling inserted in a FRAP port on the microscope. Both the TomKat dichroic and the dichroic used for myosin imaging can pass 407 nm light, enabling rapid FRAP stimulation. To focus the FRAP module, HL60 cells expressing either the TomKat FRET sensor or the mScarlet-I-Myl9 were loaded in a microfluidic device and imaged, to determine the appropriate focal plane. Cells were thereafter selected, zapped (setting the laser to 40 ms exposure and 10 % power) and imaged using the FRAP channel. The *x*-*y* translation knobs of the FRAP module were adjusted to bring the FRAP spot near the center of the image (around 512 pixel × 512 pixel on a 1024 pixel × 1024 pixel image, where 1 pixel is 0.21 μm). Thereafter, the *z*-adjustment knob was used to focus the FRAP spot into a tight approximately gaussian-shaped spot. Cell-stimulation experiments were conducted using ~1.8 μW power, as measured at the objective using a Thorlabs handheld optical power meter (PM100D) and a microscope slide power sensor (S170C). For each experimental session, we collected pictures of the FRAP spot and averaged them to identify the pixel with the maximum FRAP spot intensity. The *x*-*y* coordinates of the maximum intensity pixel were saved and later used to dynamically translate the stage, to align the maximum activation spot with the desired sub-cellular stimulation location.

Each lane of the microfluidic device was organized into a grid of *x*-*y* coordinates that were imaged consecutively. Cells were segmented in real time using a minimum fluorescence threshold and minimum and maximum size thresholds. The target point was computed by determining the left front (in the competition assays) or the retracting/losing front (in the reversal assays). Determination of the cell front relied on extracting the cell body boundary from the binary mask and determining the point in the cell perimeter as specified by a target angle (for us 0^o^ in respect to the original orientation of cell movement). For continuous and late stimulation experiments, UV stimulation and imaging were alternated until the imaging period was over. For the early stimulation assays we administered a total of 5 pulses of light. For all assays we maintained the same time interval (3 s) for imaging and stimulation. Alternating between imaging and stimulation, results in turning the receptor off with each image acquisition and then rapidly back on again with the following stimulation pulse, as parapinopsin is a G_i_α-family coupled GPCR that is activated by UV light and inactivated by orange light (> 530 nm) leading to rapid activation and deactivation cycles. Thus, our stimulation assays are pulsed.

#### Camera and illumination corrections for Cdc42 TomKat FRET and Myl9 imaging

Raw images were corrected for the camera dark-state noise, for differences in the camera chip sensitivity, and for dust in the light path as previously described^34^. In addition, a gradient in apparent FRET ratio activity was empirically observed from the top to bottom of the TomKat FRET sensor images, due to imperfections in the light path. We corrected for this gradient by developing a “ratio correction image”. For that, images of unstimulated Cdc42 TomKat FRET sensor HL60 cells loaded in microfluidic channels were collected systematically with different stage positions and the same 60x objective so that at least one cell was imaged on every portion of the camera sensor. FRET ratios were computed using our standard analysis pipeline for each image and images were assembled into a 3D image stack. To generate a single representative full-field FRET image, we computed the median of the FRET ratio over the stack (including only pixels corresponding to cells). To reduce local variability, we smoothed this image by taking the median over each 24 pixel × 24 pixel block and smoothed using a gaussian filter (sigma=5). Finally, we resized the result to generate a ratio correction image with the same dimensions as the input image. We applied the correction by dividing the FRET ratio images by the computed ratio correction image. This gradient correction was not necessary for the Myl9 imaging.

#### Cell segmentation and image background subtraction for Cdc42 TomKat FRET and Myl9 imaging

Raw Cdc42 TomKat FRET pair images were registered using the coordinate-mapping strategy described^34^. Cell segmentation and image background subtraction for the Cdc42 TomKat FRET images and the Myl9 images were performed as described before^38^. Briefly, cell segmentation was performed on the sum of the aligned FRET donor and acceptor images, to improve signal to noise ratio. We first conservatively defined background and cell object pixels and then determined a background image using the median intensity of background pixels in the local neighborhood of each pixel. We then subtracted the background image from the sum image. Object edges were enhanced by first smoothing the image using a broader gaussian filter (sigma=5), and then subtracting the smoothed image from the original image. Finally, the cell object binary masks were determined through the Otsu’s threshold method. For each FRET donor and acceptor image we subtracted the background as defined through the above segmentation strategy. Pixels not included in the cell mask were defined as not a number (NAN), to eliminate them from downstream analysis. The FRET ratio image was calculated as FRET acceptor divided by FRET donor. We used a similar strategy to register the mScarlet-I-Myl9 and cytoplasmic iRFP pair images for the myosin cell line and to subtract the background.

### QUANTIFICATION AND STATISTICAL ANALYSIS

#### Statistical learning

##### Pre-processing

Precise segmentation of cellular boundaries was critical in building the statistical learning models. Empirically, we found that manual user correction was necessary to ensure the quality of segmentation of the phase contrast images. We developed a semi-automatic workflow using the magnetic lasso and quick selection tool in Adobe Photoshop CS6 (Adobe Systems Inc.) to enable fast manual segmentation.

After defining background and cell object pixels, we calculated the median intensity of background pixels in the local neighborhood of the cell for all fluorescence images. The background was subtracted from the raw fluorescence images and fluorescence intensity was normalized across movies. All images were centered at the cell centroid and cropped to a consistent size (601 pixels × 601 pixels).

##### Feature extraction

For each cell decision-making movie, we found the image frames from when the cell first contacted the obstacle until retraction initiation. Retraction initiation was defined as the first frame where we identified a retraction area of the losing front (*i.e.*, area difference > 0). For these frames we used the open software CellProfiler 3.0 to extract an array of features from the binary masks and the fluorescence images using a custom-made image analysis pipeline. Specifically, we extracted features based on morphology (size and shape from the *MeasureObjectSizeShape* module) and intensity-based parameters (intensity statistics, texture, and granularity within segmented cells, from the *MeasureObjectIntensity*, *MeasureObjectRadialDistribution*, *MeasureTexture* and MeasureImageGranularity modules). A full list of the morphological parameters is provided in Supplemental Table 1. Our pipeline returned a total of ~700 features for each time point for further processing.

##### Post-processing

Different cells make their decisions at different rates (Supplemental Fig. 1A). To facilitate comparisons across the large population of individual cells, we filtered the data to include only events whose time from contact with the obstacle until retraction initiation was within one standard deviation of the population average (45 s ± 15 s). To synchronize the filtered decision-making events, we worked on a rescaled time, interpolating the feature values extracted from the frames of each cell decision-making movie for 15 time points between contact with the obstacle and retraction initiation, so that on average the time between two consecutive interpolated time points is ~ 3 s (equal to the frame rate of the microscopy data). Feature interpolation was carried out via fitting a cubic smooth spline with the smoothing parameter set at the default value of 0.5 (where 0 fits a straight line and 1 gives a total fit) for each cell and each feature over time.

##### Feature selection

Considering the small number of training samples in relation to the large number of extracted features (696 features extracted through CellProfiler), we predicted the binary outcome of directional decision-making using the lasso (L1 penalty) logistic regression. The lasso enables fitting a logistic regression even when the number of features exceeds the number of observations, regularizes the parameters of the logistic regression to reduce model variance, and discards features to maintain only the most predictive ones. For each dataset examined, we trained on 67 cells and tested the classifier on an independent test set of 33 cells with known labels (1 for right turning cells and 0 for left). Training and testing were performed for each time point separately. For each time point, all features were standardized by subtracting the cell average and dividing by the standard error of the mean. We cross-validated the model by implementing the cv.glmnet function with nfold=10 from the glmnet package^48^ in R. The tuning parameter λ was chosen by the 10–fold cross validation as the λ for the best model path. We used the selected model to evaluate the misclassification error in the test set. The CellProfiler features and the lasso feature coefficients across interpolated time points are presented in Supplemental Table 1. In addition to using the lasso on features extracted through CellProfiler, we also implemented the lasso on cumulative feature means as well as feature slopes computed over consequent interpolated time points, however these strategies did not improve the prediction.

We also implemented an elastic net regression and a sparse group lasso regression (Supplemental Figures 1C–1D). Elastic Net was implemented using the glmnet package; Supplemental Figure 1 illustrates the predictive accuracy, which is equal to (1-misclassification error) %, of the statistical models across interpolated time points with α=0.8 (where α=0 is ridge L2 penalty and α=1 is lasso L1 penalty). For the sparse group lasso strategy, we formed 696 feature groups, one for each feature over the 15 interpolated time points. We then applied sparse group lasso and were left with 20 groups with non-zero features. Finally, using just these 20 features we used an elastic net model with α=0.8 at each time independently and calculated the misclassification error in the test set. Even though this strategy allows the earlier time points (closer to the cell contacting the obstacle) to be informed by later ones (closer to directional decision), it showed comparable results with that of the initial lasso (Supplemental Figures 1C–1D).

#### Image analysis

##### Quantification of Cdc42 activity and myosin localization at each edge

Cell front areas were defined as the 400 pixels closest to the cell front edge. The 400 pixels enabled us to capture the entire penetration depth of the Cdc42 activity at the competing cell fronts in this channel geometry. The cell front edge point was defined as the cross section point between the midline of the channel and the cell mask in each arm of the bifurcation. We computed the Cdc42 sensor activity as the sum of the acceptor signal divided by the sum of the donor signal in that area (Figs. 4C, 4F, 4H, 4I, 5F and Supplemental Figs. 2B, 2F). Cell front Cdc42 activities were corrected for imaging-induced photobleaching using an empirically determined linear, multiplicative photobleaching correction. The photobleaching correction was computed by fitting a linear rate for the decrease in measured FRET ratio for the same Cdc42 sensor in an equal sized cell front region in well polarized cells migrating persistently without experimental perturbation in straight microfluidic channels^38^.

We used the same strategy to identify the front of Myl9-expressing cells, so that we can directly compare the dynamic re-localization of Cdc42 activity and myosin II (Supplemental Fig. 2). To analyze the relative subcellular distribution of myosin, we computed the normalized myosin at the front as the sum of the Myl9 signal in each area of interest divided by the sum of the Myl9 signal in the entire segmented cell body (Supplemental Figs. 2D, 2H). No photobleaching correction was necessary for the Myl9 signal, as photobleaching was negligible compared to the dynamic range of the Myl9 signal.

##### Cell speed, length and area quantification

To calculate the protrusion and retraction speed, we compared the 400 pixel masks every other frame, and marked the newly protruded regions and the newly retracted regions. The regions were summed up and divided by the time step in between frames to obtain the instantaneous protrusion speed and retraction speed, hence the units of speed are μm^2^/min (Figs. 4B, 4E, 5E and Supplemental Figs. 2A, 2C, 2E, 2G). Cell length was approximated via computing the Y-shaped skeleton of the binary cell mask and summing up its length segments, while cell area was calculated by summing the total number of pixels of the computed binary cell mask. Relative changes in length and area were plotted by dividing the cell length and area for each cell by its respective length and area at either the time of contact with the obstacle (Figs. 4D, 4G) or at the time when the two competing fronts had clearly separated from one another (*i.e.,* when both had extended at least 2.2 μm past the apex) (Figs. 4H, 4I).

##### Temporal cross correlation analysis

Relative change in cell length, cell stretching, was computed as described directly above, relative to cell length at the time when the two competing fronts had clearly separated from one another. The difference in front Cdc42 activity between the competing fronts was determined by subtracting the Cdc42 activity of the losing front from the Cdc42 activity of the winning front and taking the absolute value of that difference. To reduce noise, we examined traces starting at the time when the two competing fronts had clearly separated from one another and ending at the time of big retraction. In addition, we kept cells with meaningfully long traces (10 time frames, corresponding to 30 s), and excluded cells with shorter traces. These parameter traces were smoothed via a local regression using weighted linear least squares and a 2^nd^ degree polynomial model (“loess” or locally weighted smoothing in MATLAB). We used a span of 10% of the total number of data points for this smoothing. Temporal cross-correlations were then computed as a function of offset in time between cell stretching and absolute difference in Cdc42 activity between the winning and the losing cell front using Pearson’s correlation.

##### Stratification of fast and slow responding reversing cells

Computing the edge point of the retracting front as described above, we calculated the euclidean distance of that point relative to the obstacle apex point of the obstacle at two times: i) when the stimulation protocol was initiated (*i.e.* when a big retraction was detected) and we plot that on the *x*-axis of Fig. 5D, and ii) when the retracting edge had reached its minimum distance from the obstacle apex before reprotrusion commences (reprotrusion is defined as protrusion area more than 7.2 μm^2^) and we plot that on the *y*-axis of Fig. 5D. For each point, we then took the *y* coordinate and divided it by the *x* coordinate to get one number for each cell. That number should be near zero for a cell that retracts fully and near 1 for a cell that immediately reverses. We called cells for which *y*/*x* < 0.5 slow responders, and the rest fast responders.

#### Statistical analysis

Day-to-day and experiment-to-experiment variation for categorical data was consistent with Poisson counting statistics and the observed standard deviations were reasonably explained by the binomial model in each case. For quantitative signaling, speed, and localization data, cell-to-cell variability was the dominant source of variability. Given the limited and variable number of cells that could be analyzed in each experiment, we believe that individual cells represented the most relevant independent unit for statistical analysis. In addition, we performed control experiments on each day to avoid potential bias. Based on these considerations, we pooled data from all independent experiments performed. We indicate the collective number of cells per condition in the Figure Legends. All measurements were taken using independent cells. Statistical parameters and significance are reported in the Figures and the Figure Legends. Data are determined to be statistically significant when *p* < 0.05 by either two-sided Wilcoxon rank sum test or two-sided Fisher exact test (“ranksum” or “fishertest” in MATLAB, respectively).

To depict the percentage of cells across the different assays (Figs. 1B, 3C, 5B, and 5G), we represented data with bar plots showing the cumulative mean value and an error bar which represents the standard deviation assuming a binomial distribution around the cumulative mean, pooling data from all independent experiments.

For the statistical analysis of the time from front contact to (big) retraction (Fig. 4J and Supplemental Fig. 1F), the cell speed (Fig. 5E) and the subcellular localization of Cdc42 activity (Fig. 5F), we used violin plots pooling all cells that passed our pre-processing standards of masking and tracking, from all independent experiments. In each violin plot the dashed line represents the median of the distribution and the dotted lines the 25% and 75% quartiles of the distribution. To compare across conditions or cell groups, we performed non-parametric two-sided Wilcoxon rank sum tests.

#### Movie processing

Supplemental Movies 1–3 show background subtracted signals, as described above. For the Supplemental Movies 4–9, each donor and acceptor image were smoothed using a gaussian filter (sigma=2), after subtracting the background as previously described. This smoothing step was only applied to movies and not for any other analysis. The FRET ratio image was calculated as FRET acceptor divided by FRET donor (using the smoothed images). The Cdc42 activity ratio for each cell was a bit different and since the relative activity across each cell is most informative, we chose to show cells in Supplemental Movies 4–9 with a FRET range from the 1^st^ percentile to the 99^th^ percentile of the FRET ratio images of each movie.

#### Plotting

Plotting was performed using MATLAB R2018a (MATHWORKS) and GraphPad Prism 9.

## Supplementary Material

Video S1 **Supplemental Movie 1: Decision-making of an HL60 cell facing a symmetric directional dilemma.** Movie of an HL60 cell expressing actin-YFP and MRLC-mApple, representative of 164 individual cells. On the left panel we show the overlay of the phase contrast image, actin and myosin fluorescence and next to that the individual microscopy channels (in grayscale). Images were captured every 3 s. Scale bar: 10 μm.

Video S2 **Supplemental Movie 2: Decision-making of an HL60 cell facing a symmetric directional dilemma.** Movie of an HL60 cell stained with Hoechst to tag the cell nucleus and with SiR-tubulin to tag the microtubules, representative of 169 individual cells. On the left panel we show the overlay of the phase contrast image, nuclear and microtubules’ fluorescence and next to that the individual microscopy channels (in grayscale). Images were captured every 3 s. Scale bar: 10 μm.

Video S4 **Supplemental Movie 4: A cell turns left under continuous optogenetic stimulation at the left competing front.** Movie of a continuous stimulation competition assay for an HL60 cell expressing parapinopsin and the Cdc42 FRET sensor. This cell is representative of 226 left turning cells out of 304 individual cells analyzed under this assay. Initially, the cell migrated without experimental perturbation and after extending two competing fronts clearly separated from one another (*i.e.,* when both had extended at least 2.2 μm past the apex), we administered continuous optogenetic stimulation at its left competing front (magenta circle). On the left we show the registered sensors (in grayscale) and on the right the computed Cdc42 sensor activity (FRET ratio). Images were captured every 3 s. Scale bar: 25 μm.

Video S3 **Supplemental Movie 3: Decision-making of a nocodazole-treated HL60 cell facing a symmetric directional dilemma.** Movie of a nocodazole-treated HL60 cell stained with Hoechst to tag the cell nucleus and with SiR-tubulin to tag the microtubules, representative of 34 individual cells. On the left panel we show the overlay of the phase contrast image, nuclear and microtubules’ fluorescence and next to that the individual microscopy channels (in grayscale). Images were captured every 3 s. Scale bar: 10 μm.

Video S5 **Supplemental Movie 5: A cell not responding to early optogenetic stimulation at the left competing front.** Movie of an early stimulation competition assay for an HL60 cell expressing parapinopsin and the Cdc42 FRET sensor. This cell is representative of 83 right turning (non-responding) cells out of 179 individual cells analyzed under this assay. Initially, the cell migrated without experimental perturbation and after extending two competing fronts clearly separated from one another (*i.e.,* when both had extended at least 2.2 μm past the apex), we administered 5-pulses of optogenetic stimulation at its left competing front (magenta circle). On the left we show the registered sensors (in grayscale) and on the right the computed Cdc42 sensor activity. Images were captured every 3 s. Scale bar: 25 μm.

Video S6 **Supplemental Movie 6: A cell turns left under late optogenetic stimulation at the left competing front.** Movie of a late stimulation competition assay of an HL60 cell expressing parapinopsin and the Cdc42 FRET sensor. This cell is representative of 79 left turning cells out of 105 individual cells analyzed under this assay. Initially, the cell migrated without experimental perturbation and after extending two competing fronts have extended at least 6.6 μm past the apex we administered continuous optogenetic stimulation at its left competing front (magenta circle). On the left we show the registered sensors (in grayscale) and on the right the computed Cdc42 sensor activity. Images were captured every 3 s. Scale bar: 25 μm.

Video S8 **Supplemental Movie 8: A cell not reversing its direction of motion under early optogenetic stimulation at the retracting/losing front.** An unsuccessful early stimulation reversal of an HL60 cell expressing parapinopsin and the Cdc42 FRET sensor. This cell is representative of 64 non-reversing cells out of 69 individual cells analyzed under this assay. The cell migrates and decides a direction of turning without experimental perturbation. Upon identifying a big retraction, we administer 5-pulses of optogenetic stimulation (magenta circles) at the retracting/losing front. On the left we show the registered sensors (in grayscale) and on the right the computed Cdc42 sensor activity. Images were captured every 3 s. Scale bar: 25 μm.

Video S7 **Supplemental Movie 7: A cell reversing its direction of motion under continuous optogenetic stimulation at the retracting/losing front.** Movie of a successful continuous stimulation reversal of an HL60 cell expressing parapinopsin and the Cdc42 FRET sensor. This cell is representative of 66 reversing cells out of 244 individual cells analyzed under this assay. The cell migrates and decides a direction of turning without experimental perturbation. Upon identifying a big retraction, we administer continuous stimulation (magenta circles) at the retracting/losing front. On the left we show the registered sensors (in grayscale) and on the right the computed Cdc42 sensor activity. Images were captured every 3 s. Scale bar: 25 μm.

Video S9 **Supplemental Movie 9: A cell reversing its direction of motion under late optogenetic stimulation at the retracting/losing front.** A successful late stimulation reversal of an HL60 cell expressing parapinopsin and the Cdc42 FRET sensor. This cell is representative of 36 reversing cells out of 97 individual cells analyzed under this assay. The cell migrates and decides a direction of turning without experimental perturbation. We let the cell retract back to the apex of the obstacle and then began repetitively stimulating the previously retracting/losing front (magenta circles). On the left we show the registered sensors (in grayscale) and on the right the computed Cdc42 sensor activity. Images were captured every 3 s. Scale bar: 25 μm.

Supplemental Information

Supplemental Table S1 **Supplemental Table 1**: Tables of the analyzed CellProfiler features extracted from HL-60 cells undergoing symmetric decision-making. This Excel file contains two sheets. The first sheet contains the Lasso coefficients for the features extracted from the dataset composed of phase contrast images, along with fluorescence images of actin-YFP and MRLC-mApple. The second sheet contains the Lasso coefficients for the features extracted from the dataset composed of phase contrast images, along with fluorescence images of the cell nucleus (Hoechst) and microtubules (SiR-tubulin).

## HIGHLIGHTS

1. Symmetrically bifurcating microfluidic channels create competing cell fronts
2. Mechanical competition, not biochemical signaling, resolves competing cell fronts
3. Receptor inputs bias decision making only after stretching mediates competition
4. A committed retracting edge is unresponsive to receptor input until fully retracted
