# Supplementary material for: Leading edge competition promotes context-dependent responses to receptor inputs to resolve directional dilemmas in neutrophil migration
Source: Cell Syst. Author manuscript; Available in PMC 2023 May 1. (PMC10150694; doi:10.1016/j.cels.2023.02.001)
Supplement: Supplemental Information [file NIHMS1889296-supplement-Supplemental_Information.pdf]

Supplemental Information

Leading edge competition promotes context-dependent responses to receptor inputs to resolve directional dilemmas in neutrophil migration

Amalia Hadjitheodorou[1,2], George R. R. Bell[3,6], Felix Ellett[4], Daniel Irimia[4], Robert Tibshirani[5], Sean R. Collins[3,*], and Julie A. Theriot[2,7,*]

[1]Department of Bioengineering, Stanford University, Stanford, CA, USA

[2]Department of Biology and Howard Hughes Medical Institute, University of Washington, Seattle, WA, USA

[3]Department of Microbiology and Molecular Genetics, University of California, Davis, Davis, CA, USA

[4]Department of Surgery, BioMEMS Resource Center, Massachusetts General Hospital, Harvard Medical School, Boston, MA, USA

[5]Department of Statistics and Biomedical Data Science, Stanford University, Stanford, CA, USA

[6]Present address: Chan Zuckerberg Biohub, San Francisco, CA, USA

[7]Lead contact

*Correspondence: srcollins@ucdavis.edu (S.R.C), jtheriot@uw.edu (J.A.T.)

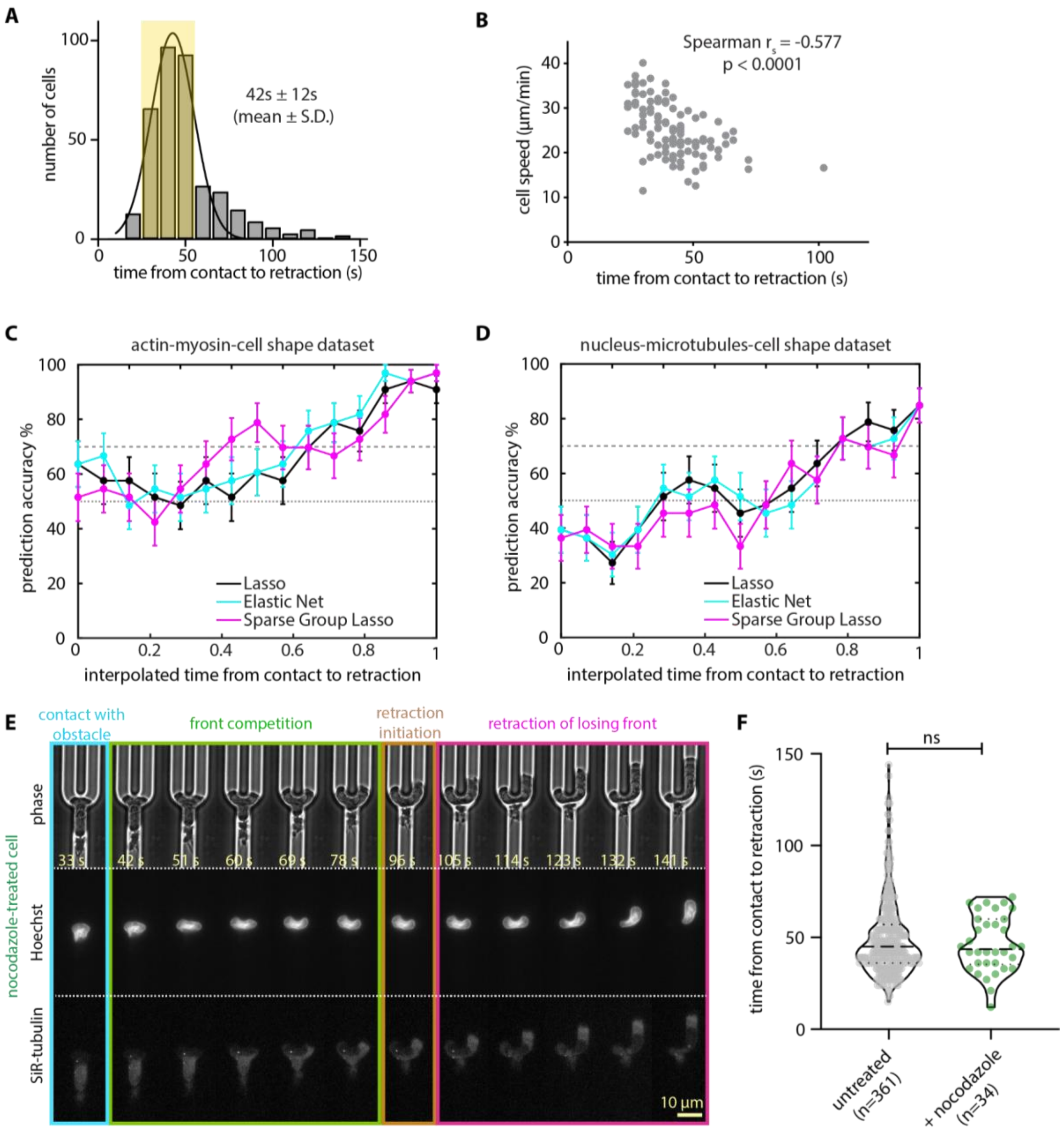

Supplemental Figure 1: Timing and predictability of cellular decisions in bifurcation devices

(A) Histogram of time from contact with the obstacle until retraction initiation for 361 cells migrating inside symmetrically bifurcating microfluidic channels. Gaussian fit yields a mean of 42 s and a S.D. of 12 s. Cells whose decision time was within one standard deviation of the mean were retained for further analysis (yellow box). **(B)** Scatter plot of average cell centroid speed before contact with the obstacle vs. time from contact with the obstacle until retraction initiation for 109 cells. Spearman correlation yields $r_s = -0.577$, p

< 0.0001. **(C-D)** Test set prediction accuracy of statistical models leveraging information contained in binary masks and fluorescence images of actin and myosin **(C)** and in binary images and fluorescence images of nucleus and microtubules **(D)** across interpolated time points between contact with the obstacle and retraction initiation. **(E)** Live-cell imaging snapshots of single HL60 cells treated with nocodazole to depolymerize the microtubules (red channel) and stained with Hoechst to tag the nucleus (cyan) during symmetric decision-making, representative of 34 individual cells (Supplementary Movie 3). Images were captured every 3 s and subsampled for illustration purposes. Scale bar: 10 μm. **(F)** Violin plot of time from contact with the obstacle to retraction initiation for 361 untreated and 34 nocodazole-treated cells; *p*-value of two-sided Wilcoxon's rank-sum test (ns: $p > 0.05$).

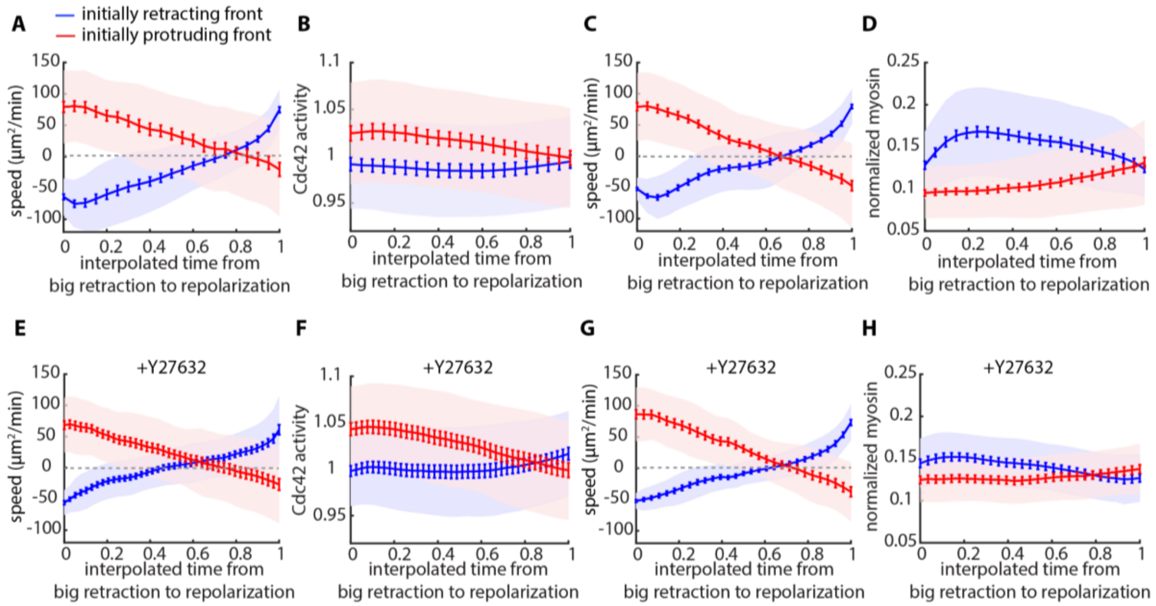

Supplemental Figure 2: Cdc42 activity and myosin II polarity reverse after directional change

Mean speed **(A)** and magnitude of Cdc42 activity **(B)** of the initially retracting (stimulated, blue) and initially protruding (red) edges for 47 cells expressing parapinopsin and Cdc42 FRET sensor that successfully reversed under the continuous stimulation reversal assay (lines: means, shaded regions: SD, error bars: mean value ± SE). Mean speed **(C)** and normalized myosin intensity **(D)** of the initially retracting (stimulated, blue) and protruding (red) edges for 70 cells expressing parapinopsin and Myl9 that successfully reversed under the continuous stimulation assay (lines: means, shaded regions: SD, error bars: mean value ± SE). Mean speed **(E)** and magnitude of Cdc42 activity **(F)** of the initially retracting (stimulated, blue) and initially protruding (red) edges for 27 Y27632-treated cells expressing parapinopsin and Cdc42 FRET sensor that successfully reversed under the continuous stimulation assay (lines: means, shaded regions: SD, error bars: mean value ± SE). Mean speed **(G)** and normalized myosin intensity **(H)** of the initially retracting (stimulated, blue) and initially protruding (red) edges for 37 Y27632-treated cells expressing parapinopsin and Myl9 that successfully reversed under the continuous stimulation assay (lines: means, shaded regions: SD, error bars: mean value ± SE).
